# Humanistic Meaning-Making and Behavioral Governance in AI-Mediated Nursing Care

**DOI:** 10.2196/103504

**Published:** 2026-11-06

**Authors:** Tino Hsieh

**Affiliations:** 1 College of Nursing, National Yang Ming Chiao Tung University, No. 155, Sec. 2, Linong St, Beitou Dist., Taipei, Taiwan, 112304, Taiwan, 886 2-2312-3456 ext 262170; 2 Department of Nursing, National Taiwan University Hospital, Taipei, Taiwan

**Keywords:** artificial intelligence, AI, nursing, digital health, nursing informatics, humanistic care, clinical judgment, behavioral governance, AI-mediated care, nursing leadership, patient recognition

## Abstract

AI has supported nursing and health care for years through decision support, prediction, monitoring, and workflow tools; what is changing is the breadth and proximity of AI-generated information to everyday care. Existing nursing AI literature has advanced AI literacy, safety, ethics, and governance, yet less attention has focused on how AI-mediated care reshapes contextual nursing judgment, patient recognition, professional behavior, and relational accountability at the point of care. Nursing digital competence may therefore increasingly need to extend beyond AI literacy toward humanistic meaning-making and behavioral governance. We offer MESH as a provisional integrative nursing lens with 4 domains: meaning-making, ethical presence, system and relational workflow governance, and human-in-the-relationship. Standardized nursing data are considered one enabling bridge through which nursing-sensitive information may remain visible within AI-supported decision environments. The MESH-action cycle—recognize, interpret, communicate, govern, and evaluate—is proposed as a 5-step practice-oriented pathway for applying these domains. MESH is not a validated competency model; its purpose is to support inquiry, education, implementation design, and empirical testing of whether AI augments or obscures nursing judgment and patient recognition.

## Introduction: AI and the Expanding Interpretive Space of Care

AI is not new to nursing or health care. Rule-based clinical decision support, automated classification, predictive models, and monitoring systems have influenced clinical work for years [1-5]. What is changing is the scope and proximity of machine-generated information to everyday care. Predictive and other AI-based technologies are increasingly used across clinical and operational functions [1,6], while generative and conversational systems can synthesize narratives, generate text, and participate more directly in clinician-patient communication [7]. AI therefore does more than add another tool to clinical workflows; it can participate in the informational and interpretive processes through which clinicians understand patients and determine care priorities.

These capabilities may improve safety, efficiency, access, and information synthesis. At the same time, as AI-enabled clinical information systems summarize, classify, and prioritize clinical information, the representation of the patient can become progressively abstracted. This raises a deeper nursing question: can care become more efficiently processed while the patient feels less personally recognized [8-10]? The concern is not limited to technical error, algorithmic bias, or inadequate literacy. It also concerns how machine-generated representations influence nursing attention, interpretation, communication, and accountability.

AI-mediated nursing care refers here to clinical situations in which AI-generated classifications, predictions, summaries, recommendations, or communications materially shape how patient information is represented, interpreted, communicated, prioritized, or acted upon within nursing workflows. It should therefore not be understood only as the use of AI tools in nursing practice. This viewpoint examines how nursing judgment, patient recognition, nursing-data visibility, professional behavior, and workflow governance interact under such conditions and proposes meaning-making, ethical presence, system and relational workflow governance, and human-in-the-relationship (MESH) as an integrative nursing lens for that interaction.

## Why AI Literacy Is Necessary but Insufficient

Recent nursing AI scholarship has advanced essential conversations across several complementary areas. AI literacy and competency work has clarified knowledge, verification, responsible use, and the capabilities nurses may need when working with AI [6,11-13]. Nursing leadership and governance scholarship has addressed implementation, sociotechnical safety, accountability, and organizational readiness [1,11,14-16]. Nursing ethics literature has emphasized dignity, respect, caring relationships, and professional accountability [9,17-19].

These contributions are necessary, but they do not fully resolve the point-of-care problem addressed here. Existing frameworks already address substantial elements of literacy, governance, communication, interpretation, and safety [1,6,11-16]. The remaining question is how these elements interact when AI becomes part of the way patients are represented, summarized, classified, and acted upon. A generated handoff note may be coherent while omitting uncertainty. A conversational AI system may provide accurate information while failing to recognize fear, shame, or confusion [7]. A staffing algorithm may optimize numbers while overlooking novice nurse support, skill mix, emotional labor, and team fatigue.

The critical question therefore shifts from “Can nurses use AI?” to “What must nursing preserve and govern when AI mediates how patients become known?” Tool use does not by itself constitute competence in AI-mediated care. Nursing competence also requires the ability to contextualize machine-generated information, recognize discordance between an output and the embodied patient, communicate uncertainty, and govern how AI changes professional behavior and clinical workflows.

## Why MESH Now? From Decision Support to AI-Mediated Care

Urgency does not arise because AI has suddenly appeared in nursing. Rather, the nature and density of mediation are expanding. Earlier clinical decision support systems often delivered bounded alerts, rules, or predictions within specific tasks; contemporary generative and conversational AI systems can also summarize narratives, draft documentation, and produce patient-facing explanations, potentially functioning as an additional informational agent within clinician-patient interactions [7]. These developments increase the number of points at which machine-generated representations can shape attention, communication, and responsibility.

The need for MESH therefore rests not on technological novelty alone but on the increasing density of AI mediation across the clinical encounter. The same human factors problems observed with earlier automation—anchoring, overreliance, misuse, disuse, and automation bias—remain relevant [4,5]. What changes is the scale at which these effects may intersect with generated language, patient communication, documentation, and workflow governance.

## Conceptual Positioning Among Existing Frameworks

In developing this viewpoint, we considered representative—not exhaustive—work in nursing AI literacy and competence, nursing AI governance, human factors, ethics, and implementation. This was a conceptual positioning exercise rather than a systematic literature search, and we do not claim to have excluded alternative frameworks through formal eligibility criteria.

Existing approaches already address substantial parts of the problem. The N.U.R.S.E.S. (navigate AI basics, utilize AI strategically, recognize AI pitfalls, skills support, ethics in action, and shape the future) guide emphasizes foundational AI literacy and responsible use [12]. NAIL-G (nursing artificial intelligence literacy and governance) integrates nursing AI literacy with sociotechnical safety and governance [11]. A modified Delphi study by Groeneveld et al [13] identified 10 competency domains for nurses working with AI-driven technologies, including interpretation of AI output, integration into clinical practice, communication, implementation, and evaluation. Human factors scholarship addresses automation bias, trust, use, misuse, and overreliance [4,5], while nursing ethics and relational scholarship emphasize dignity, respect, accountability, and the clinician-patient relationship [7-10,17,18].

MESH does not claim that these elements are absent from existing frameworks. Its proposed contribution is to bring them together around a nursing-specific question that becomes especially salient when AI mediates the point of care: how can nurses preserve contextual meaning-making and patient recognition while simultaneously governing behavioral drift, data visibility, workflow, and relational accountability? In this sense, MESH is an integrative lens for the interaction among domains that are often addressed separately, not a replacement for the frameworks from which those domains can be informed.

## Standardized Nursing Data as a Bridge Between Humanistic Judgment and AI-Supported Decision-Making

Humanistic nursing judgment should not be positioned outside the data infrastructure of AI. Nurses continuously generate clinically meaningful information through assessments of symptoms, function, behavior, coping, care dependency, patient responses, and changes that may not yet be fully represented by conventional biomedical indicators. The challenge is that much of this knowledge may remain fragmented across narrative documentation or become insufficiently represented in the structured data used for digital decision support. As AI systems increasingly depend on structured clinical information, what becomes computationally visible may influence what becomes clinically salient.

Standardized nursing diagnoses, structured nursing assessments, Nursing Minimum Data Sets, and nursing-sensitive outcomes offer possible mechanisms for making nursing knowledge more visible within digital information infrastructures. In the protocol cited here, a Nursing Minimum Data Set is used to structure nursing diagnoses, nursing actions, and dimensions of care complexity [20]. Such standardization should not be interpreted as reducing nursing judgment to codes or as evidence that structured data automatically produce better or more humanistic AI. Rather, structured nursing data may provide one informational bridge through which nursing-sensitive observations can become available for analysis while still requiring contextual interpretation.

Emerging evidence illustrates this potential. Cesare et al [21] used a machine learning approach to rank individual nursing diagnoses according to their predictive relevance for intensive care unit transfer. This finding should be interpreted cautiously: it does not establish that standardized nursing documentation automatically improves AI performance, nor does it imply that all dimensions of nursing judgment can be computationally encoded. It does show that structured nursing diagnoses may contain clinically meaningful information that is relevant to deterioration risk.

This distinction matters for humanistic nursing. Without adequate representation of nursing-sensitive information, AI systems may reproduce a predominantly biomedical view of the patient that underrepresents function, vulnerability, patient response, psychosocial concerns, care dependency, and other dimensions routinely recognized by nurses. Standardized nursing data are therefore treated in MESH as an enabling informational bridge—particularly for meaning-making and system and relational workflow governance—not as an additional framework domain. The relevant question is not whether nursing judgment should be human or data driven but how nursing knowledge can remain clinically meaningful, contextually interpreted, and professionally governed as portions of that knowledge become structured and computationally available.

## When Machine-Generated Interpretation and the Patient Do Not Match

The following hypothetical emergency department vignette is clinically grounded and is intended to illustrate a class of machine-mediated interpretation errors rather than describe an identifiable patient.

An automated cardiac monitoring system displays a tachycardic rate. At the bedside, however, the nurse palpates a substantially slower pulse. Rather than accepting the displayed rate as definitive, the nurse reassesses the patient, reviews the waveform, obtains a more detailed medication history, and escalates the discordance for specialist review. Hospital-based continuous electrocardiogram monitoring systems can generate false-positive arrhythmia alarms, underscoring the need to interpret monitor output in conjunction with the patient and waveform rather than in isolation [22]. In this hypothetical scenario, the displayed tachycardic rate is recognized as a monitoring artifact rather than the patient’s true pulse rate, while the actual bradycardia is clinically consistent with exposure to a glaucoma medication capable of systemic beta-blocking effects [23].

The clinical value of nursing judgment in this scenario lies not in rejecting technology but in recognizing that the machine-generated representation and the embodied patient do not correspond. Uncritical acceptance of the displayed rate could have prompted inappropriate tachyarrhythmia-directed evaluation or treatment. The monitor supplied a machine-generated representation; the nurse recognized the discrepancy and reconstructed its clinical meaning.

Conventional cardiac monitoring is not equivalent to contemporary generative AI. The vignette is used as an analogue of a broader machine-patient discordance problem rather than as evidence that all automated monitoring is AI. Across clinical technologies, machine-generated classifications can acquire cognitive prominence once integrated into clinical workflows. Human oversight is therefore necessary but not sufficient. Nursing requires accountable human presence: examining the patient, interpreting the clinical context, listening to the patient's story, communicating uncertainty, and taking responsibility when a technology-generated representation does not adequately reflect the person receiving care.

## AI-Mediated Behavioral Drift

AI implementation can change more than information flow. As with earlier forms of automation, AI-enabled systems may alter attention, trust, questioning, communication, documentation, escalation, and responsibility [4,5]. These changes may occur gradually after implementation and may not be captured by conventional metrics such as adoption, usability, accuracy, or time saved.

We use the term “AI-mediated behavioral drift” provisionally to describe gradual, normalized postimplementation shifts across multiple professional behaviors—including attention, trust, communication, documentation, escalation, and accountability—rather than a single decision error or a synonym for automation bias. Automation bias concerns overreliance on automated output in judgment or decision-making [4]; misuse and disuse describe inappropriate patterns of automation use [5]. Behavioral drift, as proposed here, concerns how multiple AI-influenced behaviors may become normalized within clinical workflows over time. Examples may include looking first at a risk score rather than at the patient’s embodied presentation, reproducing AI-generated summaries without sufficient contextual revision, or making disagreement with AI outputs difficult to document.

Such drift is not inevitable, but it warrants governance attention. In this viewpoint, behavioral governance does not mean disciplinary monitoring of individual nurses. It refers to governance of AI- and workflow-induced changes in professional attention, trust, communication, documentation, escalation, workflow, and accountability through AI system design, feedback loops, escalation structures, team learning, and nonpunitive review. Humanistic meaning-making requires calibrated trust: nurses need to judge when an output is clinically plausible, contextually incomplete, ethically concerning, or operationally useful [3-5]. Recognition of AI-clinical discordance—the mismatch between machine-generated output and bedside assessment—is therefore proposed as a candidate observable safety behavior rather than an informal act of professional intuition.

## The MESH Framework

To move this discussion from abstract concern to practice-oriented inquiry, we offer MESH as a provisional conceptual framework for humanistic meaning-making and behavioral governance in AI-mediated nursing care. MESH denotes 4 domains—meaning-making, ethical presence, system and relational workflow governance, and human-in-the-relationship—and should not be confused with Medical Subject Headings (MeSH). In this domain, “system” refers to the sociotechnical health care environment and unit-level workflow in which AI is implemented, rather than only to an electronic health record system. MESH is offered in conversation with existing nursing AI literacy, competence, leadership, and governance frameworks [11-14]. It is not presented as a validated theory, complete competency model, or exhaustive taxonomy.

Meaning-making refers to the nursing capacity to interpret AI outputs through patient narratives, embodied assessment, family concerns, clinical trajectory, structured nursing data, and available resources. Ethical presence concerns the moral obligations that remain active during AI-mediated care, including dignity, autonomy, equity, advocacy, vulnerability, uncertainty, and moral accountability [8-10,17,18]. System and relational workflow governance concerns how AI system design, health care leadership, and unit-level workflow shape documentation, escalation, responsibility, invisible labor, and relational burden. Human-in-the-relationship concerns accountable human relational presence: the nurse remains responsible for listening, contextualizing, explaining uncertainty, responding to patient and family questions, supporting shared understanding, and ensuring that automated communication does not substitute for therapeutic interaction.

MESH is not intended to replace existing AI literacy, competency, ethics, leadership, or governance approaches [6,11-15,17-19], nor does it claim novelty for its component concepts. Its proposed contribution is their point-of-care integration around AI-mediated patient representation, nursing meaning-making, behavioral adaptation, nursing-data visibility, workflow governance, and relational accountability, together with translation of these concerns into provisional targets for education, implementation, audit, and empirical study.

## What MESH Adds to Existing Frameworks

MESH does not aim to replace established nursing theories, AI competency frameworks, implementation science, human factors models, or approaches to AI literacy. Each contributes important concepts for understanding professional judgment, technology adoption, workflow, safety, ethics, and person-centered care [4,5,11-14]. The distinctive purpose of MESH is narrower: to organize these concerns around the conditions created when machine-generated information becomes an active intermediary in nursing care.

In AI-mediated practice, interpretation, relational recognition, behavioral adaptation, data representation, and workflow governance do not occur independently. An AI-generated recommendation may simultaneously redirect a nurse’s attention, influence how a patient is interpreted, alter communication, modify escalation behavior, and redistribute accountability within workflow. Frameworks designed primarily to explain technology adoption, professional competency, ethical principles, or health care technology implementation may illuminate individual dimensions of this process without making their interaction at the point of nursing care explicit.

MESH therefore functions as an integrative nursing lens rather than a competing grand theory. Its contribution lies in connecting 4 concurrent questions: What does the AI output mean in this patient’s context? What moral concerns and dimensions of the person remain visible? How is the technology shaping professional behavior, data visibility, and workflow? And who remains relationally present and accountable when nurses, patients, families, and AI-generated information interact? Its proposed contribution lies in this point-of-care integration and in translating these questions into observable targets for education, implementation, audit, and empirical research.

## Operationalizing MESH in Clinical Practice

The practical value of MESH depends on whether its domains can be observed, taught, audited, and empirically examined. Table 1 therefore links each domain to a clinical question, observable indicators, implementation examples, and potential research directions. The indicators are intentionally provisional; they are not presented as validated measures. Rather, they illustrate how concepts such as patient recognition and behavioral drift can be converted into testable implementation constructs.

**Table 1.** Operationalizing the MESH (meaning-making, ethical presence, system and relational workflow governance, and human-in-the-relationship) framework in AI-mediated nursing care.

MESH domain	Key clinical question	Observable indicators	Example implementation	Future empirical direction
Meaning-making	Does the AI output improve or distort the interpretation of this patient’s situation?	Nurse compares output with bedside assessment, patient narrative, trajectory, family context, and structured nursing information; AI-clinical discordance is recognized and investigated as a candidate safety behavior.	A cardiac monitor displays a tachycardic rate that conflicts with the palpated pulse; the nurse reassesses, reviews the waveform and medication history, and escalates the discrepancy rather than treating the machine-generated display as definitive.	Candidate indicators: frequency and resolution of AI-clinical discordance, override patterns, missed contextual cues, and escalation consequences.
Ethical presence	Does AI-mediated care preserve moral obligations related to dignity, autonomy, equity, advocacy, vulnerability, and uncertainty?	Patient preferences, vulnerability, goals, concerns, and ethically relevant uncertainty remain visible in documentation and decision-making.	AI-generated discharge information is reviewed and adapted to the patient’s comprehension, concerns, family or community support, and ability to self-manage.	Candidate indicators: omission of preference-sensitive information, perceived respect, trust, and individualized care measures.
System and relational workflow governance	How does AI alter attention, escalation, accountability, workload, documentation, and relational work?	Defined escalation and override pathways, audit trail, alert and documentation burden monitoring, frontline feedback, and nonpunitive review of recurrent AI-influenced workflow problems.	A unit reviews recurrent overrides, alert fatigue, delayed responses, documentation revisions, and examples of behavioral drift at regular governance meetings.	Candidate indicators: alert fatigue, automation bias, behavioral drift, escalation performance, workload, and documentation burden.
Human-in-the-relationship	Does AI augment rather than substitute accountable human relational presence?	Nurse listens, contextualizes, explains uncertainty, responds to questions, supports shared understanding, and remains accountable for communication.	AI prepares preliminary education or summarizes information; the nurse verifies it, explains uncertainty, and contextualizes it through conversation and teach-back.	Candidate indicators: patient comprehension, shared understanding, teach-back performance, trust, and perceived relational quality.

## The MESH-Action Cycle

A conceptual lens that identifies a problem but does not support application is of limited value for nursing leadership. We therefore pair the 4 MESH domains with a 5-step MESH-action cycle: recognize, interpret, communicate, govern, and evaluate. MESH identifies what should be examined and preserved; the action cycle is a practice-oriented application pathway for beginning that work, not a second framework or theory. Each step draws on established bodies of knowledge. Figure 1 illustrates the relationship between the four MESH domains and the MESH-action cycle. Table 2 summarizes the five steps of the MESH-action cycle, including the corresponding leadership actions, intended purposes, and supporting knowledge bases.

**Table 2.** MESH (meaning-making, ethical presence, system and relational workflow governance, and human-in-the-relationship)-action cycle for nurse leaders.

Action	Nurse leaders should...	Purpose	Starting knowledge base
Recognize	Identify how AI reshapes attention, trust, communication, documentation, data visibility, and accountability	Detect behavioral drift and representational gaps early	Human factors, usability, and safety science [4,5]
Interpret	Train nurses to compare AI outputs with bedside assessment, patient narratives, family concerns, structured nursing data, and clinical trajectory	Prevent automation bias, premature closure, and unexamined discordance	Clinical reasoning, automation bias, calibrated trust, and AI competency work [3-5,13]
Communicate	Prepare nurses to explain AI-supported care and uncertainty in language that preserves trust and understanding	Maintain patient and family recognition and participation	Therapeutic and relational communication, respect, and shared understanding [7-10,13]
Govern	Establish escalation, override, documentation, and feedback pathways when AI and nursing judgment diverge	Protect accountability, safety, and professional agency	Sociotechnical safety, implementation science, and nursing leadership and governance [1,11,14,15]
Evaluate	Use nursing-sensitive, behavioral, relational, and humanistic indicators in addition to accuracy, adoption, and efficiency metrics	Ensure AI-supported care remains clinically meaningful and humane	Nursing-sensitive outcomes and implementation evaluation [1,13,15,16]; human factors and automation research [4,5]

**Figure 1.**
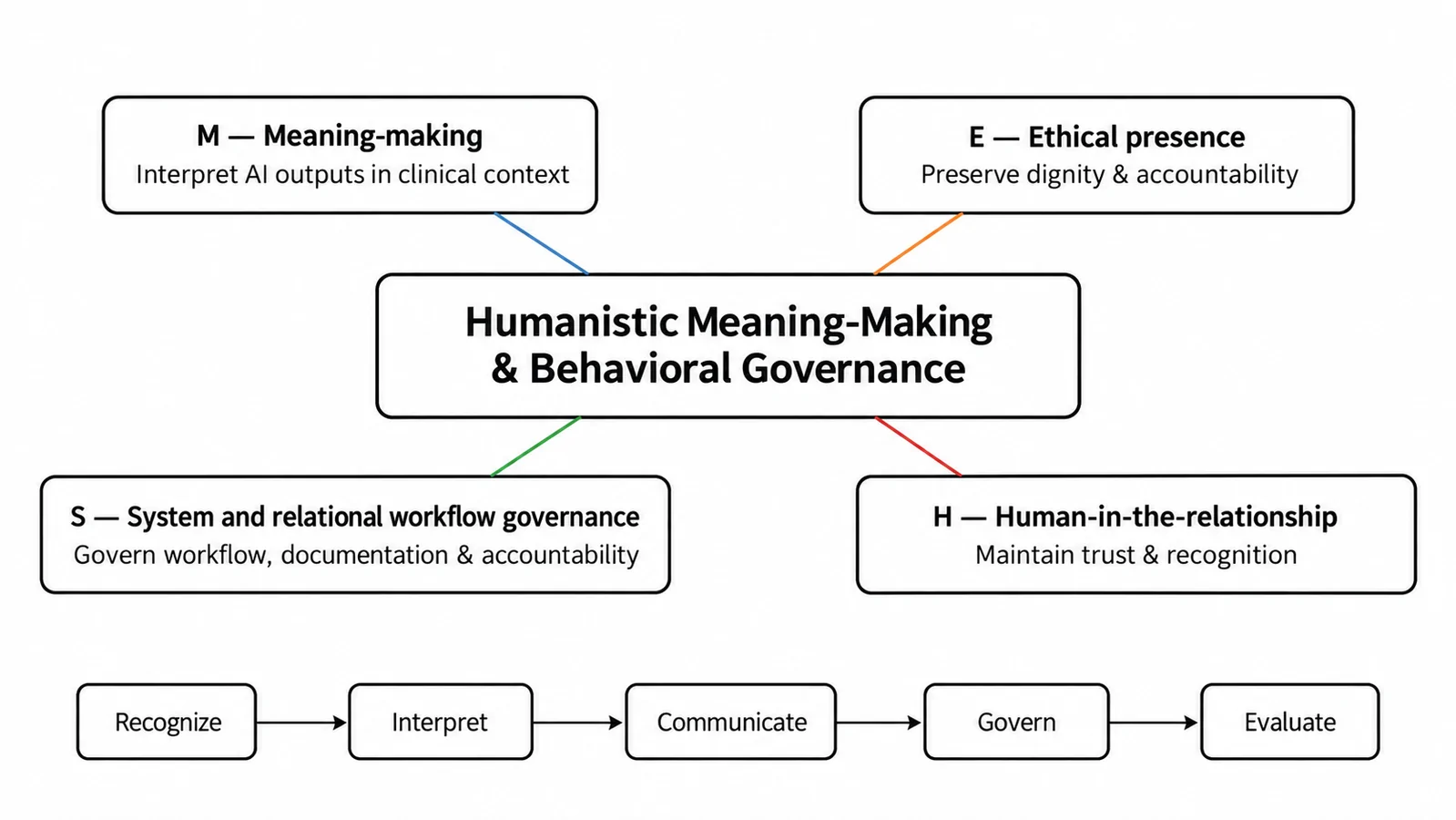
The MESH (meaning-making, ethical presence, system and relational workflow governance, and human-in-the-relationship) framework and MESH-action cycle for AI-mediated nursing care. The 4 MESH domains identify what nursing should examine and preserve in AI-mediated care, whereas the 5-step MESH-action cycle provides a practice-oriented pathway for applying these domains in leadership, education, implementation, and evaluation; it is not a second framework or theory.

“Recognize” can draw on human factors and safety science to identify AI-induced changes in attention, trust, communication, data visibility, workflow, and accountability before they become normalized [4,5]. “Interpret” builds on clinical reasoning, automation bias research, calibrated trust, and AI competency work to reconcile machine output with the embodied patient, narrative, nursing data, trajectory, and context [3-5,13]. “Communicate” focuses on AI-supported information, uncertainty, therapeutic communication, and shared understanding [7-10,13]. “Govern” draws on sociotechnical safety, implementation, and nursing leadership scholarship to establish escalation, override, accountability, audit trails, and feedback systems [1,11,14,15]. “Evaluate” extends technical performance assessment to candidate nursing-sensitive, behavioral, relational, workload, and patient-experience consequences [1,4,5,13,15,16].

Potential indicators include AI-nursing judgment discordance, documented rationales for overriding AI recommendations, alert burden, AI-generated documentation revision rates, patient understanding of AI-supported care, family communication concerns, staff confidence, missed care, perceived moral distress, and nursing-sensitive outcomes. These indicators do not replace technical evaluation; they broaden evaluation to the human and professional consequences of implementation.

## AI as an Enabler and Risk Amplifier of Humanistic Care

AI should not be framed only as a threat to humanistic nursing. Reviews of AI-based technologies in nursing describe applications in decision support, prediction, education, and workflow-related functions [1,6,16]. Generative and conversational systems may additionally support information synthesis and communication [7]. Whether these functions actually reduce cognitive or administrative burden or create more time for relational care is an empirical question rather than an assumed benefit. Under appropriate conditions, however, AI may support rather than diminish humanistic care.

At the same time, the humanistic consequences of AI are not inherent properties of the technology; they are produced through its design, data, interpretation, implementation, and governance. The same AI-enabled clinical information system that surfaces a clinically meaningful pattern can also promote automation bias; the same generated summary that reduces documentation effort can narrow a clinical narrative; and the same decision support tool that standardizes care can shift responsibility or normalize behavioral drift [3-5,7].

The relevant distinction is therefore not humanistic nursing vs AI but humanistically governed AI vs poorly governed AI-mediated care. AI outputs should function as inputs into nursing meaning-making, not as substitutes for it. The objective of governance is to preserve the benefits of computational support while maintaining contextual judgment, patient recognition, and professional accountability.

## Professional Advocacy and Leadership Implications

Nursing should not be positioned merely as the workforce that adapts to AI-enabled technologies after they are designed, purchased, and implemented [1,14,15,17,18]. Nurses and nurse leaders are governance actors because AI-enabled clinical information systems can alter clinical attention, documentation, escalation, workload, patient communication, and the visibility of nursing knowledge.

At the unit level, MESH may guide organizations to examine more than technical infrastructure and staff training. Candidate governance questions include where AI-generated information enters clinical workflows, what nursing-sensitive information is included or omitted, who remains accountable when AI and bedside assessment diverge, whether escalation and override pathways are available, how alert and documentation burden change, and whether frontline feedback identifies recurrent mismatches between AI-enabled clinical information system outputs and clinical reality. These questions are intended for organizational learning rather than individual surveillance or blame.

For nursing education, MESH may extend AI literacy toward AI-mediated clinical reasoning [4-6,12]. Simulation could expose learners to plausible but incomplete outputs, conflicting bedside findings, and situations in which AI-generated communication requires contextual revision. Training can cultivate calibrated trust rather than blind compliance or defensive rejection.

For research, validation should proceed in stages rather than assume that MESH improves outcomes. Initial qualitative work and stakeholder consultation could refine domain boundaries and language. Delphi studies could then assess content relevance and clarity among frontline nurses, nurse leaders, informaticians, patients, and ethicists. Subsequent work could develop candidate indicators or audit items and test them in simulation or observational studies, including recognition of AI-clinical discordance and override behavior. Only after conceptual and operational refinement should implementation studies examine feasibility, acceptability, workflow effects, patient experience, and associations with nursing-sensitive outcomes.

## Limitations and Future Validation

The MESH framework is proposed as a provisional conceptual framework rather than an empirically validated model. Its purpose is to organize emerging concerns in AI-mediated nursing care and generate practice-oriented questions for education, leadership, implementation, and research. We do not claim that the 4 domains are exhaustive, universally applicable, psychometrically established, or unique in their component concepts.

The framework’s proposed contribution is integrative rather than exclusive. The conceptual positioning in this viewpoint was based on a nonsystematic comparison of representative frameworks and was not intended to establish that MESH uniquely incorporates concepts absent from all previous models. Future work should test whether its domains are distinct, useful, measurable, and applicable across clinical contexts. The proposed operational indicators require further empirical development and validation, while the relationship between standardized nursing data and AI-supported decision-making warrants investigation beyond its early applications in predictive modeling. MESH should therefore be understood as a starting language for inquiry and governance, not as a final competency standard.

## Conclusions

AI will increasingly shape nursing practice, but the central issue is not whether nursing becomes more or less technological. It is whether nursing judgment, nursing-sensitive information, patient recognition, and professional accountability remain visible and actionable within technology-mediated care. Existing AI literacy and governance frameworks provide an essential foundation [6,11,12,14,17-19]; an important next task is to examine what happens when AI enters not only the workflow but also the process through which patients become known [7-10].

To move from general readiness to practice-level evaluation, nursing needs a structured way to examine how AI-mediated care affects interpretation, patient recognition, workflow, and relationships at the point of care. This viewpoint offers MESH as a provisional integrative nursing lens and the MESH-action cycle as a practice-oriented application pathway for leadership, education, implementation, and research. The central claim is not that nursing should resist AI or that MESH introduces unprecedented component concepts. Its proposed value lies in integrating nursing meaning-making, ethical presence, nursing-data visibility, AI-induced behavioral and workflow change, and accountable human presence at the point where AI mediates how the patient is represented and understood.

When AI helps the health care system detect patterns in patient data, nursing must ensure that the patient remains recognized as a person whose experiences, vulnerabilities, preferences, and context matter.

## Abbreviations

MeSH: Medical Subject Headings
MESH: meaning-making, ethical presence, system and relational workflow governance, and human-in-the-relationship
N.U.R.S.E.S: navigate AI basics, utilize AI strategically, recognize AI pitfalls, skills support, ethics in action, and shape the future
NAIL-G: nursing artificial intelligence literacy and governance

## References

[1] von Gerich H, Moen H, Block LJ (2022). Artificial intelligence -based technologies in nursing: a scoping literature review of the evidence. Int J Nurs Stud.

[2] Topol EJ (2019). High-performance medicine: the convergence of human and artificial intelligence. Nat Med.

[3] Char DS, Shah NH, Magnus D (2018). Implementing machine learning in health care—addressing ethical challenges. N Engl J Med.

[4] Goddard K, Roudsari A, Wyatt JC (2012). Automation bias: a systematic review of frequency, effect mediators, and mitigators. J Am Med Inform Assoc.

[5] Parasuraman R, Riley V (1997). Humans and automation: use, misuse, disuse, abuse. Hum Factors.

[6] El Arab RA, Al Moosa OA, Abuadas FH, Somerville J (2025). The role of AI in nursing education and practice: umbrella review. J Med Internet Res.

[7] Campos HO, Wolfe D, Luan H, Sim I (2025). Generative AI as third agent: large language models and the transformation of the clinician-patient relationship. J Particip Med.

[8] Sim I, Cassel C (2024). The ethics of relational AI—expanding and implementing the Belmont principles. N Engl J Med.

[9] Paladino MS (2025). Artificial intelligence in nursing care: a reflection from the ethics of care. Nurs Ethics.

[10] Beach MC, Duggan PS, Cassel CK, Geller G (2007). What does “respect” mean? Exploring the moral obligation of health professionals to respect patients. J Gen Intern Med.

[11] Ibrahim FM, Shahrour G, Abuijlan IA, Abdallah AM, Alsuwayed A, Alanazi AR (2026). NAIL-G: a conceptual best-fit framework for nursing AI literacy and governance anchored in socio-technical safety science. BMC Nurs.

[12] Hoelscher SH, Pugh A (2025). N.U.R.S.E.S. embracing artificial intelligence: a guide to artificial intelligence literacy for the nursing profession. Nurs Outlook.

[13] Groeneveld SW, van Os-Medendorp H, van Gemert-Pijnen JE (2025). Essential competencies of nurses working with AI-driven lifestyle monitoring in long-term care: a modified Delphi study. Nurse Educ Today.

[14] Burford JS, Booth RG, McIntyre A (2025). Exploring the intersection of nursing leadership and artificial intelligence: scoping review. JMIR Nurs.

[15] Ronquillo CE, Peltonen LM, Pruinelli L (2021). Artificial intelligence in nursing: priorities and opportunities from an international invitational think-tank of the Nursing and Artificial Intelligence Leadership Collaborative. J Adv Nurs.

[16] Buchanan C, Howitt ML, Wilson R, Booth RG, Risling T, Bamford M (2020). Predicted influences of artificial intelligence on the domains of nursing: scoping review. JMIR Nurs.

[17] Stokes L (2025). ANA position statement: the ethical use of artificial intelligence in nursing practice. Online J Issues Nurs.

[18] (2025). 2025 code of ethics for nurses provision 7.5: considerations related to ethics, technology, and policy. American Nurses Association.

[19] Jobin A, Ienca M, Vayena E (2019). The global landscape of AI ethics guidelines. Nat Mach Intell.

[20] Cesare M, D’Agostino F, Cocchieri A (2024). Exploring the association between complexity of care, medical complexity, and length of stay in the paediatric setting using a Nursing Minimum Data Set: a study protocol. Nurs Rep.

[21] Cesare M, Nurchis MC, Damiani G, Cocchieri A, Nursing and Public Health Group (2025). Ranking nursing diagnoses by predictive relevance for intensive care unit transfer risk in adult and pediatric patients: a machine learning approach with random forest. Healthcare (Basel).

[22] Cosentino N, Zhang X, Farrar EJ (2024). Performance comparison of 6 in-hospital patient monitoring systems in the detection and alarm of ventricular cardiac arrhythmias. Cardiovasc Digit Health J.

[23] Pratt NL, Ramsay EN, Kalisch Ellett LM, Nguyen TA, Roughead EE (2015). Association between ophthalmic timolol and hospitalisation for bradycardia. J Ophthalmol.

